# Adapting eating disorder treatment for ADHD: clinicians’ perspectives on building structure, harnessing strengths, and negotiating boundaries

**DOI:** 10.1186/s40337-026-01740-5

**Published:** 2026-08-19

**Authors:** Lauren Makin, Valeria Mondelli, Kate Tchanturia

**Affiliations:** 1 Department of Psychological Medicine, Institute of Psychiatry, Psychology & Neuroscience, King’s College London, PO59, 16 De Crespigny Park, London, SE5 8AF UK; 2 National Institute for Health and Care Research (NIHR) Maudsley Biomedical Research Centre at South London and Maudsley NHS Foundation Trust and King’s College London, London, UK; 3 Department of Eating Disorders, South London and Maudsley NHS foundation Trust, London, UK; 4 Department of Culture and Communication, Aalborg University, Aalborg, Denmark

**Keywords:** ADHD, Eating disorders, Neurodiversity, Qualitative, Clinician, Reflexive thematic analysis, Treatment, Multidisciplinary

## Abstract

**Background:**

ADHD is overrepresented in clinical eating disorder (ED) services, and patients with ADHD present to services with increased ED psychopathology, psychological distress, and functional difficulties. They are also more likely to drop out of treatment and have poorer outcomes. Although previous studies have explored patients with ADHD’s perspectives on their ED and treatment, clinician accounts remain underexplored, despite clinicians shaping recognition, formulation, and treatment adaptation in routine care. This study therefore explored ED clinicians’ perspectives on working with patients with ADHD.

**Methods:**

Semi-structured interviews were conducted with 19 multidisciplinary ED clinicians and analysed using reflexive thematic analysis through a neuro-affirming lens.

**Results:**

Three themes were developed: (1) *ADHD as pervasive life chaos*; (2) *Harnessing energy*,* alliance*,* and creativity*; and (3) *Navigating service boundaries*.

**Conclusions:**

Clinicians described ADHD-related disorganisation, reward-seeking, and emotional volatility as underpinning ED behaviours and disrupting therapeutic processes, highlighting the importance of supporting structure and planning during treatment. Strength-based, collaborative, and creative approaches were also emphasised. However, challenges around recognising, diagnosing, and medicating ADHD were particularly salient, highlighting the need for greater collaboration and knowledge exchange between ED and ADHD services, alongside better use of existing multidisciplinary and dual expertise within ED teams.

**Supplementary Information:**

The online version contains supplementary material available at https://doi.org/10.1186/s40337-026-01740-5.

## Abstract (plain language summary)

ADHD is more common in people receiving treatment for eating disorders than in the general population. People with co-occurring ADHD and eating disorders often experience more severe symptoms and poorer treatment outcomes. While some research has explored the experiences of people with co-occurring ADHD and eating disorders, less is known about clinicians’ experiences of working with this group. This study interviewed 19 clinicians from different professional backgrounds working in eating disorder services (for example psychiatrists and dieticians). The interviews explored their experiences of supporting patients with ADHD. Three main findings were identified. First, clinicians described ADHD as creating difficulties with organisation, emotions, and daily routines, which could affect eating behaviours and make treatment harder to follow. Supporting structure, routines, and emotional stability was therefore seen as important for recovery. Second, clinicians highlighted strengths often associated with ADHD, such as energy, social skills, and creativity, and described working collaboratively and flexibly with patients to use these strengths in treatment. Third, clinicians reported limited communication between eating disorder and ADHD services, creating challenges around ADHD diagnosis and medication. Overall, the findings suggest that stronger collaboration within eating disorder teams and between eating disorder and ADHD services may help improve care for this group.

**Supplementary Information:**

The online version contains supplementary material available at https://doi.org/10.1186/s40337-026-01740-5.

## Background

‘Attention-deficit/hyperactivity disorder’ (ADHD) is a neurodevelopmental difference associated with distinct patterns of attention, activity regulation, and impulsivity, which vary across individuals and contexts [1]. These patterns reflect differences in planning, attention regulation, reward sensitivity, emotional regulation, and sensory processing. Such differences may influence eating behaviours, vulnerability to eating disorders (EDs), and engagement with treatment. Although ADHD has long been studied in relation to impulsivity, binge eating, and co-occurring psychiatric conditions [2–6], it has historically received less attention within ED services than Autism and has rarely been examined using an explicitly neuro-affirming lens in this context [7].

Contemporary neurodiversity-informed frameworks increasingly conceptualise both Autism and ADHD as minoritised neurotypes, associated with increased risk of misrecognition, stigma, and insufficient accommodation within healthcare systems [8, 9]. This reconceptualization has coincided with growing recognition of Autism and ADHD in adult populations, particularly among women [10, 11]. Conceptual development of neuro-affirming ED care has been largely driven by Autism research [12], which has highlighted the roles of environmental mismatch, sensory overwhelm and masking (camouflaging) in ED vulnerability [13–24]. However, ADHD has been underexplored in this area.

ADHD is over-represented among individuals with EDs compared to the general population. A meta-analytic review estimated that ADHD is approximately twice as common in people with EDs [25], although this association is likely to have increased post-COVID [26]. Within specialist ED services, prevalence estimates are notably high. Approximately one-third of adults attending specialist ED services screen positive for probable ADHD [27]. Reported ADHD prevalence varies substantially by ED diagnosis and subtype. Estimates range from 3 to 35% in anorexia nervosa (AN), 9–58% in bulimia nervosa (BN), 10–67% in binge ED (BED), and approximately 29% in ED not otherwise specified (EDNOS) [7, 25, 27, 28]. Subtype analyses suggest higher ADHD prevalence in binge-purge presentations compared with restrictive presentations of AN and EDNOS [27]. Emerging work also extends beyond traditional ED diagnoses: approximately one quarter of individuals with avoidant/restrictive food-intake disorder meet criteria for ADHD [29], and a recent biobank study found elevated odds of ADHD among individuals with night eating syndrome [30].

Despite this elevated prevalence, ADHD is often overlooked during treatment and failure to recognise ADHD within ED services has emerged as a significant concern for patients. Qualitative studies indicate that individuals with suspected or diagnosed ADHD often report uncertainty about whether their ADHD was relevant to their ED or treatment and wish this had been explored during care [23, 24]. For those who accessed diagnosis and psychoeducation, recognition of ADHD was described as facilitating self-understanding, reducing self-stigma, and supporting coping [23, 24]. Furthermore, participants with co-occurring ADHD and long-term EDs reported that ADHD traits were misattributed to personality pathology or resistance to treatment, and that ADHD assessment was deferred until ED symptoms were considered resolved [31].

ADHD is also associated with greater needs within ED services. Patients with co-occurring ADHD and EDs show increased ED psychopathology, and increased anxiety and depression symptoms compared to those with EDs but without ADHD [27, 32–34]. ADHD has also been associated with increased dropout rates from ED treatment and increased ED symptoms post-treatment [35, 36]. When asked what would help them engage more effectively in treatment, patients with ADHD report wanting more structured sessions during ED treatment and support with building routines, alongside flexibility regarding attendance and between-session tasks [23, 24].

However, there are still many gaps in our understanding of presentation and treatment needs of ADHD in ED services, despite improving ED treatment outcomes being a community-identified research priority [37]. In particular, clinicians’ experiences of working with patients with co-occurring ADHD and EDs are important as clinicians shape recognition, formulation, treatment adaptions, and ultimately patient outcomes. They also offer a unique vantage point: unlike patients or carers, clinicians can compare across multiple presentations with and without ADHD, and unlike researchers, they work with individuals directly within real-world clinical contexts. However, to our knowledge, only one study has looked at the perspective of clinicians on ADHD in EDs. In a study of fourteen BED experts (researchers and clinicians), most participants (*n* = 13) viewed ADHD as relevant to BED, citing frequent co-occurrence, possible mechanisms, diagnostic challenges, and mixed views on the use of stimulant medications and other treatments [38].

Consequently, we currently know very little about how clinicians manage ADHD in ED services. The present study therefore aims to explore clinicians’ perspectives on working with patients with co-occurring ADHD and EDs, with particular attention to treatment approaches and needs.

## Methodology

This qualitative study used semi-structured interviews and reflexive thematic analysis [39, 40] to explore perspectives of clinicians on working with patients with co-occurring ADHD and EDs. NHS ethical approval was obtained (REC: 24/LO/0573).

The study was underpinned by a critical realist framework informed by the neurodiversity paradigm [8, 41–44]. This approach assumes that neurodevelopmental differences are real, while recognising that their meanings, interpretations, and consequences are shaped by social and clinical contexts. Consistent with this position, we conceptualised ADHD as involving differences that interact with environmental demands, rather than as inherent deficits. Reflexive thematic analysis was chosen as it allows rich exploration of experiential accounts and clinician meaning-making.

Throughout this paper we use person-first language (“people with ADHD”) because current evidence suggests this is preferred by most UK adults with ADHD [45]. However, participant quotations retain original terminology.

### Context & procedure

Participants were eligible if they were clinicians working within a specialist ED service. Previous clinician qualitative studies on Neurodivergence in EDs have typically included 9–19 participants across varied professional backgrounds [13, 14, 16, 38, 46–49]. A target sample of up to 20 was therefore selected to capture disciplinary diversity while remaining manageable for in-depth analysis, as saturation is not a goal of reflexive thematic analysis [50]. Recruitment targeted clinicians likely to have experience of and/or interest in ADHD and Neurodivergence. A list of potential participants was developed by the first author, the principal investigator, and a collaborating clinician. Psychologists/psychotherapists (including clinical psychologists, counselling psychologists, assistant psychologists, CBT practitioners, art therapists, and family therapists), as well as psychiatrists, occupational therapists, and dietitians were targeted. Consistent with reflexive thematic analysis, sampling aimed to generate information-rich accounts from clinicians with relevant experience rather than to achieve representativeness (purposive sampling), with recognition that participants’ service contexts would shape meaning-making and were therefore analytically relevant.

Forty UK-based clinicians were invited via email, and interviews were scheduled on a first-response basis until the target group size was reached. Participants received study materials via email, including the participant information sheet, consent form, and a questionnaire, prior to the interview. Interviews were conducted by the first author between August and November 2025. Participants could choose to meet in person or on video call; most opted for video (65%). Although interviews could be split across two sessions, all were completed in one session. Interviews lasted on average 40 min (range = 20–60 min). Data analysis started after all data had been collected.

The interviews formed part of a larger project examining clinician perspectives on Autism and ADHD in ED services. Approximately half of each interview focused on Autism within binge-type EDs [51], with the remainder addressing ADHD across ED diagnoses. Twenty clinicians were interviewed in total. One recording was unusable due to technical difficulties. Nineteen interviews therefore were analysed.

### Measures

#### Background questionnaire

The demographic information (e.g., age, gender, ethnicity, Neurodivergent identification), professional background (job role, discipline, years of experience, ED experience, service setting), and experience working with Autistic patients and those with ADHD, including self-rated confidence and previous training were obtained with questionnaire prior to the interview.

#### Interview schedule (see supplementary materials appendix 1)

A semi-structured interview schedule was developed to explore clinicians’ knowledge and perspectives regarding patients with ADHD within ED services. Questions focused on awareness and recognition of ADHD, perceived challenges and strengths when working with this population, and adaptations to treatment. Clinicians were also asked about training needs, available resources, and service-level factors influencing care for patients with co-occurring ADHD and EDs.

### Data analysis

Interviews were audio-recorded, automatically transcribed, manually checked for accuracy, and anonymised by the first author. They were stored securely in password-protected systems in accordance with institutional data management policies.

The first author conducted the reflexive thematic analysis using Microsoft Word, moving semi-iteratively between data familiarisation, coding, and theme development [39]. Coding attended both to explicit meanings and to underlying assumptions shaping clinicians’ accounts. Emerging interpretations were discussed in academic forums, including presentations at the Wales Autism Research Centre, to support reflexive consideration of alternative readings and to enhance analytic depth. Once preliminary themes were developed, analysis was moved to NVivo 15 [52], to support aggregation of relevant quotes and to support further theme refinement.

Consistent with reflexive thematic analysis, transcripts were treated not as direct windows into objective reality but as accounts shaped by context, professional culture, and broader systems of meaning. Clinicians’ descriptions of patients therefore provided insight into how ADHD is understood and enacted within services. Interpretation was informed by neurodiversity-affirmative scholarship and models frequently used within contemporary neurodiversity research, such as the emotional burden model [53, 54]. These frameworks acted as sensitising concepts that supported analysis while remaining open to divergence and complexity within the data. For example, where accounts emphasised difficulties such as emotional dysregulation, we also considered interpretations that located these experiences within unmet needs, cumulative emotional burden, or service-context demands. This approach shaped the analytic emphasis towards interactional and contextual explanations rather than purely trait-based explanations.

### Positionality and reflexivity

Reflexive positioning is reported for authors directly involved in data analysis and interpretation (LM) and supervisory interpretation of analytic development (KT).

LM is a PhD researcher specialising in Autism, ADHD, and ED research. In keeping with reflexive practice, she considered how her academic position, commitment to neurodiversity-affirmative care, and areas of shared understanding with participants may have shaped data generation, interpretation, and analytic focus. Rather than striving for neutrality, these positions were acknowledged and used as analytic resources.

KT is a Professor of Psychology and Consultant Clinical Psychologist within an ED service. She has longstanding clinical and research interests in improving treatment experiences for Neurodivergent individuals within ED services. KT pioneered the PEACE pathway [55] and adjunct interventions for EDs, and maintains ongoing collaboration with clinicians, individuals with lived experience, and carers. The study aims were therefore informed by both stakeholder feedback and KT’s clinical and research expertise.

The research team adopted a critical realist stance within a neurodiversity framework, with the explicit aim of generating knowledge that could contribute to improving ED services for people with ADHD [37]. Throughout analysis, attention was paid to how these theoretical and professional positions shaped interpretation.

### Findings

### Participant characteristics

Nineteen clinicians were included in the analysis. Participants median age was 40 (IQR = 32–43) and were predominantly female (84%) and white (79%). Around 60% were from London-based NHS trusts and the rest were from around the UK. Professional backgrounds included psychology (Clinical and Assistant) and psychotherapy (including art therapists, CBT therapists, and family therapists), psychiatry, dietetics, nursing, and occupational therapy. Several participants worked across multiple service types, professional backgrounds, or age groups. Clinical experience varied, with around half having more than 10 years of overall clinical experience and around half having more than 5 years of ED-specific experience. Most participants worked in outpatient services, although some worked in day or inpatient settings. Participants worked with adults, children and adolescents, or both, although this information was not available for all participants. Most clinicians reported experience working with patients with ADHD, but only one had received ADHD-specific training. Self-reported confidence in working with ADHD varied from “not at all confident” to “quite confident”, with “somewhat confident” being the most common response; no participant described themselves as “very confident”. A small number of participants reported having ADHD themselves, although this information was not available for all participants.

### Reflexive thematic analysis findings

**Fig. 1:**
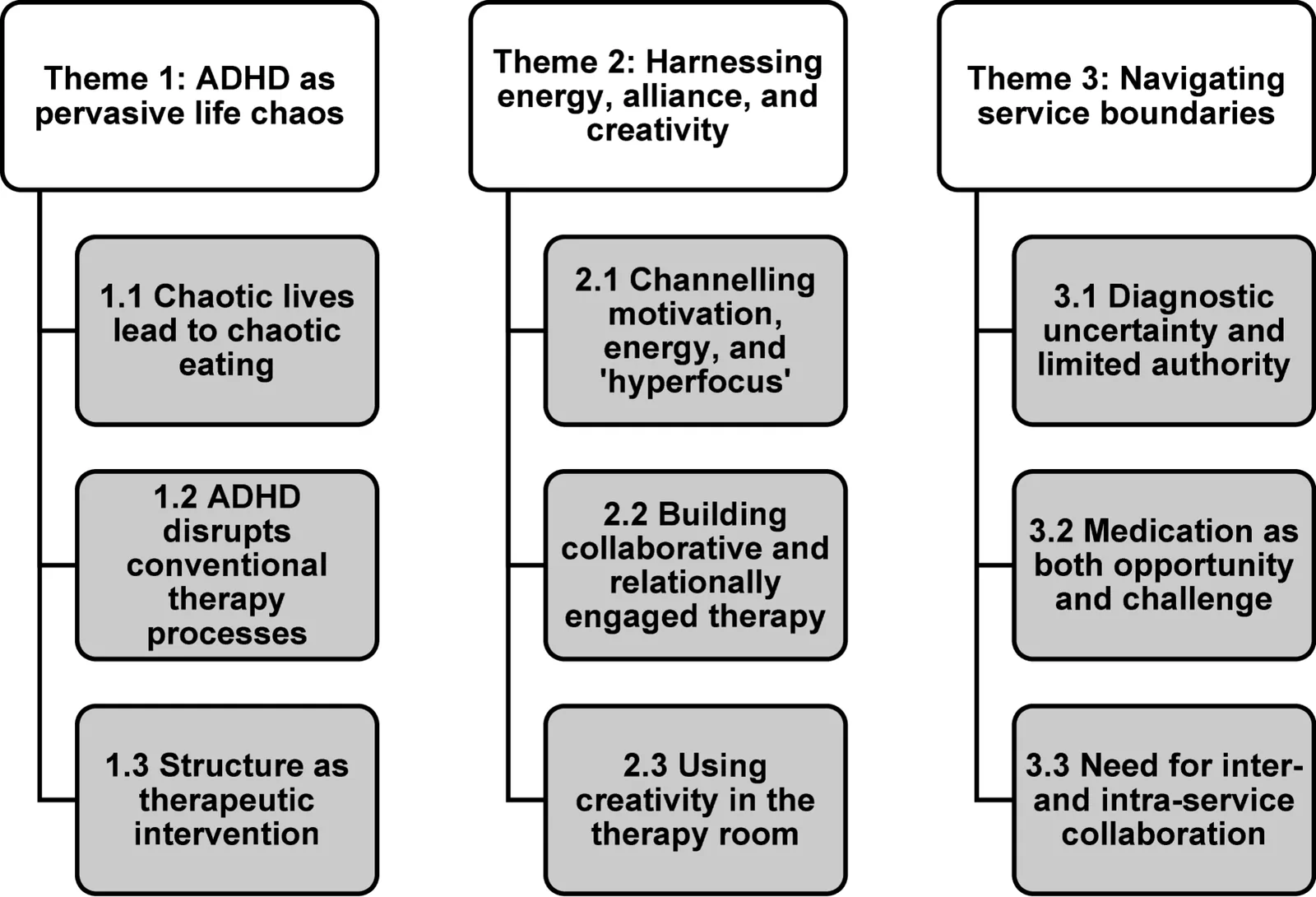
The developed themes and sub-themes.

Participants discussed their perspectives on working with patients with co-occurring ADHD and EDs. Three themes were developed (see Fig. 1).

### Theme 1: ADHD as pervasive life chaos

Clinicians described how ADHD-related disorganisation, reward-seeking, and emotional volatility underpinned ED development and maintenance in this population, as well as disrupting conventional ED therapy processes. However, they also outlined how structure and therapeutic containment could be used to manage these disruptions.

1.1 *Chaotic lives lead to chaotic eating*.

Clinicians commonly described ADHD as creating broader patterns of disorganisation and instability in patients’ daily lives, within which ED behaviours emerged. Difficulties with planning, time management, and attention were understood to contribute to irregular or *“chaotic”* [001, Psychology; 006, Dual-Role] eating patterns, including forgetting to eat, long gaps between meals, or impulsive bingeing once hunger intensified. Chaotic eating was therefore seen as reflecting wider difficulties maintaining routine and structure.

*“[That’s] one of the things that […] is contributing to their eating disorder where they’re not able to hold that structure” – 001*,* Psychology*.

Binge-type behaviours were sometimes understood as driven by impulsivity, reward sensitivity, or sensory seeking, whereas restriction was interpreted as serving a regulatory function, helping individuals create a sense of order or control within otherwise chaotic internal environments. Overexercising was similarly interpreted as a form of self-regulation in the context of hyperactivity.

*“There’s also a very big element of seeking something specific*,* like dopamine or specific sensory sensations” – 017*,* Psychology*.

*“The anorexia […] comes in because you’re trying to get some order for the ADHD” – 011*,* Dual-Role*.

Clinicians situated these challenges alongside emotional volatility and longstanding unmet needs. Patients were described as experiencing intense, rapidly shifting emotions *(“strong*,* strong feelings”* [002, Psychology]), alongside heightened rejection sensitivity and low self-esteem, which could also contribute to carer and family burnout. Growing up with unrecognised ADHD was seen as fostering chronic feelings of failure or being misunderstood, shaping both emotional wellbeing and treatment engagement.

*“The impact of going through school or growing up in a family where you didn’t understand the world […] has such an enormous impact [on] your internal self-criticism and relationships” – 004*,* Psychology*.

1.2 *ADHD disrupts therapy processes*.

Clinicians described ADHD traits as affecting the structure and pace of psychological therapy, with attention and conversation often moving unpredictably. Discussions could shift rapidly between topics, patients might become distracted, and clinicians sometimes needed to repeat themselves. Although this could make sessions feel unfocused, clinicians noted that patients were often still engaged, even if their attention appeared elsewhere.

*“[Therapy session] can just go off on a bit of a direction that’s not expected or unrelated to what the initial session plan was. So that can be hard to kind of bring it back into focus.” – 015*,* Occupational*.

*“She was really focused on the doorknob in the room. But at the same time*,* she was listening.” – 002*,* Psychology*.

Executive functioning difficulties also affected engagement with core elements of ED treatment, such as keeping food diaries, attending appointments, or completing between-session “homework”. Patients could be highly motivated in sessions, but follow-through was described as inconsistent, leading to feelings of shame in patients and frustration for clinicians. These behaviours could also easily be misinterpreted as disengagement or lack of motivation, when instead they often reflected underlying ADHD-related barriers such as forgetfulness, overwhelm, or difficulties with planning and habit formation. Similar challenges could arise when working with children or adolescents whose carers also had ADHD.

*“Sometimes there’s a lot of positive motivation and good intention in the room*,* but that doesn’t always translate to the next session [where they’ll say] oh*,* I did it for a day and I didn’t do it again.” – 002*,* Psychology*.

*“People who maybe we’ve lost from treatment in the past*,* we’d call them non-completers or disengaging*,* maybe it’s actually just really hard for them to get themselves to sessions. Both remembering the appointment*,* getting on the train in time*,* all of that.” – 004*,* Psychology*.

1.3 *Structure as therapeutic intervention*.

Clinicians felt standard ED treatments stabilising eating and emotion were appropriate for this population, but additional focus on meal planning and external structure was crucial. Strategies included setting alarms, scheduling shopping and meal prep, and building daily routines like walks or fixed therapy times. Reducing cognitive load was also important, for example using microwave meals, breakfast bars, or breaking tasks into smaller steps. Regular eating acted as an *“anchor”* [016, Psychology] within otherwise chaotic daily lives, and improvements in structure were thought to reduce ED behaviours. Occupational therapists felt particularly well placed to support patients in developing practical skills related to planning, food preparation, and independent living. However, clinicians also stressed the need for realistic expectations, noting that some irregularity in eating patterns is likely.

*“Planning might not come very easily to them*,* so maybe thinking about more instructive ways of doing planning*,* maybe giving them a template and getting them to do things like that might help.” – 009*,* Dietics*.

*“Having the snack prepared so that it’s already beside you*,* so if you do become very fixated on the task*,* it’s much easier to reach beside you than go to the kitchen*,* prepare it*,* stop the task.” – 017*,* Psychology*.

Clinicians also described actively structuring therapy to support patients with ADHD. Strategies included agenda-setting, summarising key points, and preparing patients for transitions. A more directive stance was often used, redirecting conversations when attention drifted and maintaining focus on goals. Written summaries, reminders for between-session tasks, and proactive appointment reminders helped provide external structure that patients struggled to generate independently. Some clinicians also set firm boundaries, such as refusing to start sessions if patients were very late or had not completed homework. However, most stressed that flexibility was key, with structure and containment as therapeutic tools.

*“So*,* if we start suddenly getting into some other topic*,* which often I find happens*,* I’d probably interrupt and say it sounds like we’re getting on to a new area*,* I know this is important*,* how about we make a note of this and we pick it up next time? I don’t think we’ve got time to do it justice” – 002*,* Psychology*.

### Theme 2: harnessing energy, alliance, and creativity

Alongside the challenges described in Theme 1, clinicians also emphasised that ADHD traits could bring significant strengths to treatment and recovery. Clinicians often aimed to harness them, adapting treatment collaboratively to suit the patient’s style of thinking and engaging.

2.1. *Channelling motivation*,* energy*,* and ‘hyperfocus’*.

Many clinicians described patients with ADHD as bringing high energy, enthusiasm, and strong motivation into therapy. Patients generated many ideas and showed a desire to act and progress quickly. When this energy was directed towards recovery goals, clinicians felt it could become a powerful therapeutic asset. Similarly, ‘hyperfocus’ was also seen as beneficial, as when engaged in a task or goal, patients could show remarkable persistence and commitment. Clinicians adapted sessions to work with this momentum, using shorter, focused sessions, or making the most of periods of sustained attention.

*“They have a lot of energy and drive. And if that can be put towards something positive like recovery*,* then that’s really positive.” – 014*,* Psychology*.

*“If the hyperfocus ends up working to your advantage […] they really get going or care enough about a therapy task or a goal […] it can be amazing.” – 004*,* Psychology*.

*“If I’ve only got 15 minutes of their attention*,* I have to utilise that as best as possible.” – 007*,* Dietics*.

2.2 *Building collaborative and relationally engaged therapy*.

Clinicians highlighted that ADHD traits could support a strong therapeutic alliance. Patients were often described as talkative, expressive, and socially engaged, providing rich material for therapeutic work. Additionally, their rapid idea generation and openness could help sessions progress quickly, revealing patterns and experiences that could then be explored therapeutically. Many patients were relatively self-aware about their needs, enabling more collaborative approaches. Clinicians emphasised jointly adapting treatment strategies, drawing on patients’ knowledge of what had worked previously. ADHD also seemed to support engagement in peer-based or group approaches, with shared experience fostering mutual support.

*“We will say […] these are the kinds of things we could do*,* but what do you think would make this feel helpful for you?” – 003*,* Psychology*.

2.3 *Using creativity in the therapy room*.

Clinicians also described many patients with ADHD as highly creative and resourceful, and this was often incorporated into therapeutic work. Visual tools, brainstorming, diagrams, and colour-coded materials were used to make content more engaging and accessible. Art therapy was particularly valued, allowing patients to express experiences difficult to articulate verbally through drawing, painting, or other visual methods. Sensory or movement-based elements were also incorporated, including walking during sessions, sitting outside, doodling, fidget objects, or tossing a ball while talking. Practical adaptations such as sensory screening, communication passports, and tailored monitoring tools helped clinicians better understand patients’ needs and adjust treatment accordingly. Harnessing creativity and sensory engagement supported expression, maintained attention, and made therapy more accessible and meaningful for patients with ADHD.

*“Painting a picture helps people with ADHD more than a conversation […] they can show the strength of their emotions better.” – 008*,* Psychiatry*.

*“We might walk around in the garden*,* we might sit outside*,* […] have fidget toys […]*,* or pen and paper to doodle.” – 003*,* Psychology*.

### Theme 3: navigating service boundaries

Lastly, clinicians highlighted that the siloing of ED and ADHD services reduced confidence in both their own and other services’ decisions around ADHD diagnosis and medication, underscoring the need for greater knowledge exchange, shared care planning, and better use of multidisciplinary and lived-experience expertise.

3.1. *Diagnostic uncertainty and limited authority*.

Clinicians described substantial uncertainty when recognising or discussing ADHD within ED services. Routine screening was often absent, and ED-related behaviours, such as restlessness, hyperactivity, or memory difficulties, could be misattributed to ADHD or vice versa. Clinician confidence varied, with some clinicians feeling ADHD was outside their expertise or service remit. In contrast, clinicians with lived experience of ADHD reported that their insights were often undervalued, with team disagreements occurring. Stigma, such as perceptions of ADHD as an “everyman disorder” [011, Dual-Role], and patient reluctance further complicated discussions. As a result, ADHD was often framed less as a formal diagnosis and more as a shared understanding to guide treatment.

*“You don’t pick up ADHD in those settings*,* which is why I don’t think as clinicians we can rely on what someone looks like in the room. We need to be thinking but what would they look like in a classroom? What would they look like if this was a boring situation?” – 014*,* Psychology*.

However, access to ADHD services was also challenging. Clinicians often left it to patients to explore the possibility of ADHD or pursue a diagnosis via their GP, rather than actively supporting them through the process. While some clinicians could refer patients directly to specialist ADHD services, this was uncommon. Long waiting lists frequently meant patients were discharged from ED services before receiving an assessment, which clinicians felt could undermine treatment if unrecognised ADHD was contributing to ED behaviours or limiting engagement. Private assessments were felt to carry risks of overdiagnosis and commercial bias, and ADHD services were perceived as having limited awareness of EDs.

*“There is a slight incentive to make a positive diagnosis*,* [private ADHD services] have been doing fairly dodgy assessments […] certain NHS services won’t accept those results.” – 012*,* Psychiatry*.

3.2 *Medication as both opportunity and challenge*.

Relatedly, medication emerged as another area where service boundaries became particularly visible. Clinicians viewed ADHD medication as potentially improving engagement with treatment, including attendance and implementation of coping strategies, and in some cases reducing bingeing or purging. However, concerns were raised around appetite suppression, cardiovascular effects, and the perception that ADHD services did not always account for patients’ EDs when prescribing. Most ED clinicians did not feel confident prescribing themselves, citing limited training, uncertainty around titration and monitoring, and responsibility for ongoing prescribing after discharge. Publicly funded services also faced questions about which service would hold prescribing responsibility and costs. Consequently, patients often had to access ADHD services for medication, where long waiting lists frequently delayed treatment for months or years, limiting their ability to fully engage with ED care.

*“I have spoken to the ADHD service and asked people to be put back on meds even though they’re a very low BMI because […] they were just so chaotic*,* they weren’t able to use the therapy at all” – 012*,* Psychiatrist*.

*“[ED] teams hesitate to prescribe ADHD medication because it’s not part of their usual remit. Meanwhile*,* long waiting lists for [ADHD services] mean patients can’t be seen for months or even years. […] even if a psychiatrist in the team is willing to prescribe and the family supports it*,* when the ED treatment ends*,* who takes over? GPs are often reluctant” – 003*,* Psychology*.

3.3 *Need for inter- and intra-service collaboration*.

Given these challenges, clinicians emphasised the need for stronger links between ED and ADHD services, and within ED teams. Suggested approaches included joint care pathways, shared case discussions, in-house referrals, and collaborative medication reviews. Training and knowledge exchange were seen as essential, particularly given that ADHD had received less attention than autism within ED services. Collaboration within ED services was also emphasised, with multidisciplinary teams, including occupational therapists, dietitians, art therapists, and clinicians with lived ADHD experience, playing key roles in developing ADHD-informed adaptations. Thus, addressing the challenges of ADHD within ED treatment was often framed not as the responsibility of a single clinician or service, but as requiring broader system-level collaboration and learning.

*“If we had access to some kind of joint clinic where our patients could get sort of fast tracked*,* […] that would be really good because then you can have those joint discussions” – 012*,* Psychiatry*.

*“How important linking up and professional liaison is for patients who might find that really hard to advocate for themselves with […] managing lots of different appointments [and] lots of different services.” – 019*,* Psychology*.

## Discussion

This study explored clinician’ perspectives on ADHD in EDs. ADHD-related difficulties with maintaining routine and structure were seen as underpinning their ED development and maintenance, alongside other factors like reward-seeking and emotional volatility. ADHD traits were also seen to disrupt psychological therapy, through affecting the structure and pace of sessions and affecting attendance and task completion. Thus, clinicians felt that helping patients with planning and structure was a key part of therapy. Clinicians also described working collaboratively with patients with ADHD to adapt therapy in ways that was helpful for the patient, drawing on this population’s high energy, enthusiasm, and motivation, as well as their idea generation and creativity, to build strong therapeutic alliance and experiment together in finding effective methods for implementing change. Multidisciplinary involvement was seen as key here, with occupational therapists being highly knowledgeable in improving structure, planning and other important life skills, and art therapists as being important for enabling patients with ADHD to utilise their creativity and express themselves non-verbally.

However, clinicians varied with how willing or confident they felt in recognising ADHD in patients and bringing this up with them, often seeing this as out of their remit while working in an ED service, and clinicians with ADHD felt that their lived experience was sometimes seen to invalidate their opinion despite feeling more confident and knowledgeable in this area. However, ED clinicians were concerned that ADHD services had long waiting times and limited consideration of EDs when making diagnostic and treatment decisions. This issue was particularly salient for decisions about medication, where ED clinicians felt unable to make prescription decisions themselves, but felt that ADHD medication may be helpful for getting patients to engage in ED treatment and in reducing some binge and/or purge behaviours. However, they also had concerns about the risk of these medications in reducing appetite or having adverse cardiac effects on patients who were underweight or had restrictive EDs, and they did not think these were always sufficiently considered by clinicians working in ADHD services. Clinicians felt that increased knowledge exchange between these services would help when making clinical decisions for this patient group, as well as reducing demand on the patients themselves to manage multiple services.

### Clinician versus patient accounts

Clinician accounts in this study largely aligned with previous research on patient perspectives as well as previous accounts of BED researchers and clinicians [38](see Table 2), which is interesting given that most clinicians had not received any formal training in ADHD. Four qualitative studies have included the experiences of participants with co-occurring ADHD and EDs [23, 24, 31, 56]. Across these studies, patients reported that ADHD was often undiagnosed or that assessment was deferred, and many wished the possibility of ADHD had been raised and discussed during their ED treatment [23, 24, 31]. Patients also described disorganisation, reward-seeking (and some sensory seeking), and emotional volatility as underpinning their ED [23, 24, 56], consistent with clinician perspectives. Medication was sometimes reported to negatively affect eating [24], although medication appeared less salient in patient accounts than in clinician accounts. Patients also expressed concern that their ADHD-related difficulties may be misinterpreted as ‘treatment resistance’ [31], although they reflected less on how ADHD disrupted treatment processes, possibly due to differences in study aims [23, 24, 31, 56]. Nonetheless, helpful supports included structured eating and treatment, external prompts, and strategies to reduce cognitive load [23, 24]. Patients also valued flexible, collaborative care; building trust; and peer support [23, 24], all in line with clinician reports.

**Table 2:** Comparison of findings across patient and clinician studies regarding ADHD in EDs

Topic	Clinician perspectives (this study; 38)	Patient perspectives (23, 24, 31, 56)	Integration of current paper with existing literature
Recognition, medication, and service collaboration	ADHD often missed; concerns about ADHD services’ ED awareness; medication is both opportunity and challenge; need for inter- and intra-service collaboration	ADHD often undiagnosed or deferred; desired discussion during ED treatment; medication noted as affecting eating	Both groups report ADHD often missed and services lack integration; medication concerns mainly raised by clinicians
Underlying mechanisms	ADHD-related disorganisation, reward-seeking, and emotional volatility underpin the ED; Sensory seeking; restrictive eating conceptualised as a strategy to regain control or manage emotional burden and unmet needs	ADHD-related disorganisation, reward-seeking, and emotional volatility underpin the ED; some sensory seeking and sensory sensitivities; restriction described as a means of control; additional identity-related factors (e.g., gender identity)	Clinicians and patients largely agree on how ADHD traits contribute to EDs; patients additionally emphasise broader identity-related contributors
Effect on treatment	Attention shifts, inconsistent follow-through, sometimes misinterpreted as disengagement	ADHD traits may be seen as ‘treatment resistance’	Both groups note risk of misattribution; patients less focused on treatment disruption
Need for structure	Emphasis on planning, structure, and reducing cognitive load	External prompts/structure and reduced cognitive load around eating and treatment helpful	Both agree structure and planning support is important
Strength-based, collaborative care	ADHD traits can support engagement; creative/non-verbal techniques and peer support help	Valued flexible, collaborative care, trust-building, and peer support	Both support strengths-based, collaborative approaches

### Clinicians’ perceptions of maintaining mechanisms

Both clinicians and patients suggested that ADHD traits may underpin ED behaviours in patients with ADHD [23, 24]. However, a recent scoping review found that commonly studied ADHD traits (inattention, hyperactivity, and impulsivity) do not appear to explain a total or causal relationship, highlighting the role of additional or alternative mechanisms [57].

Reward-seeking was frequently described as contributing to binge eating [23, 24, 56], and is supported by quantitative evidence suggesting shared genetic liability between ADHD, binge eating, and overeating via dopaminergic pathways [57]. Sensory seeking was also raised by clinicians and patients [23, 24, 56], although its role in EDs has not yet been explored quantitatively.

Emotional volatility and unmet emotional needs were also described as important mechanisms. Participants linked these to broader identity and self-esteem difficulties associated with unrecognised or unaffirmed ADHD [23, 24], as well as non-ADHD related identity factors [56]. Individuals with ADHD experience more frequent emotionally upsetting events than peers without ADHD, which may interact with heightened emotional intensity to increase cumulative emotional burden [53, 54]. Within this framework, eating behaviours may function as accessible strategies for regulating overwhelm.

Supporting this model, a lived-experience-led narrative review highlighted the broader social and identity-related contributors to ED risk in ADHD, including stigma, masking, trauma, and minority stress [20]. Quantitative evidence also supports this pathway: emotional dysregulation, anxiety, and depression seem to mediate associations between ADHD traits and disordered eating [57], with mood and anxiety symptoms accounting for an estimated 57–93% of the ADHD-ED association [58].

There are several alternative explanations for the association between ADHD and EDs. ED symptoms themselves may exacerbate ADHD traits, supported by evidence that elevated ADHD traits decrease following recovery from an ED but persist among those who do not recover [59]. Conversely, ADHD traits and ED symptoms may interact reciprocally over time, each exacerbating the other. Alternatively, both may arise from shared underlying vulnerabilities, such as dopaminergic mechanisms [57]. Current evidence is insufficient to distinguish between these possibilities. Nevertheless, understanding how clinicians, patients, and researchers conceptualise these potential mechanisms may help inform future hypothesis generation and treatment development.

### Clinicians’ understanding of treatment needs

Overall, this study suggests that clinicians’ understanding of the needs of patients with co-occurring ADHD and EDs largely aligns with patients’ self-reported needs including support with structure and planning, structured and boundaried treatment, and care that is collaborative, creative, and flexible [23, 24, 31]. Clinicians’ recognition of strengths associated with ADHD also aligns with emerging research emphasising strengths such as creativity, hyperfocus, and socio-affective skills [60–62].

Clinicians’ and patients’ emphasis on organisation, planning, and time-management skills aligns with broader evidence on interventions for ADHD. For example, a recent comparison of behavioural therapy packages for adolescents with ADHD identified these skills as core elements across interventions [63]. Skill application assignments, progress monitoring, and the use of rewards were also commonly included [63], which may be particularly relevant given the reward sensitivity described in ADHD-ED populations [23, 24, 57]. Many of these elements overlap with existing ED treatments, suggesting potential opportunities for adapting current approaches rather than developing entirely new interventions.

However, two key areas of concern emerged: recognition of ADHD and the use of ADHD medication. Both this study and patient accounts suggest that ADHD remains under-recognised within ED services, despite the availability of screening tools such as the Adult ADHD Self-Report Scale (ASRS) [64], which has demonstrated reliability in ED populations [65] and is widely used in ED research [27, 32, 35, 36, 59, 66]. The Eating Disorders Clinical Research Network is establishing a UK-wide research infrastructure across child and adult ED services [28]. As part of this initiative, a core dataset including optional ADHD screening via the ASRS is being introduced, representing a step toward more standardised assessment.

ADHD medication also emerged as a significant area of concern for ED clinicians. Evidence regarding its use in ED populations is emerging but remains limited. A recent scoping review summarised an open trial of lisdexamfetamine in BED, in which individuals with ADHD showed clinically meaningful reductions in binge frequency, alongside case reports suggesting potential benefits of stimulant medication for BN and BED [7]. However, adverse effects, including appetite suppression and weight loss, have been reported in isolated cases [7]. A more recent observational study suggests that ADHD medication may improve ADHD-related difficulties without worsening ED behaviours or weight outcomes in individuals with restrictive eating histories [67]. A new scoping review is currently underway to examine potential side effects of ADHD medication in ED populations more systematically [68].

More broadly, these findings highlight the consequences of siloed ED and ADHD services, with clinicians calling for greater knowledge exchange between them. Only 32% of ED clinics report having shared care protocols for neurodivergence [69], and it remains unclear how often these include ADHD. Greater communication, collaboration, and shared care planning between services are therefore needed, alongside better use of expertise within multidisciplinary teams. This includes drawing on occupational therapists to support skills related to structure, planning, cooking, and independent living, as well as sensory screening and adaptations; art therapists to facilitate non-verbal and creative approaches; and clinicians with lived experience or dual expertise. Strengthening these connections may improve recognition of ADHD within ED services and support more coordinated, effective care for patients with co-occurring ADHD and EDs.

### Strengths, limitations, and future directions

This is the first in-depth qualitative study exploring clinicians’ perspectives on ADHD in ED services and included a multidisciplinary clinical group. It addresses an important gap in a literature that has predominantly focused on the prevalence, clinical associations, and outcomes, rather than clinicians’ perspectives on formulation and treatment, which are central to improving patient experiences and outcomes. The study recruited UK clinicians involved in or interested in ADHD-informed ED care. This likely enhanced the depth and specificity of insights, as participants were more aware of ADHD within their clinical populations. However, perspectives may reflect professionals already engaged with neurodiversity-informed approaches, and findings may not transfer to clinicians less interested in ADHD.

Just over half of participants were clinical psychologists or psychological therapists (e.g., CBT therapists or assistant psychologists), meaning psychotherapy perspectives were particularly prominent compared with disciplines such as dietetics or occupational therapy. However, this reflects the central role of psychological therapies within ED services, and the relatively large participant group (for a qualitative study) strengthens the breadth of perspectives captured.

This study focused solely on ADHD, despite Autism and ADHD frequently co-occurring in the general population [70], and both being overrepresented in ED populations [7, 71, 72]. Emerging evidence also suggests that AuDHD individuals may experience greater psychological distress and functional difficulties, compared to Autistic-only or ADHD-only patients with EDs [34], indicating potential additive risk.

Future research should examine the role of sensory seeking in ADHD-ED populations, evaluate treatment adaptions that support structure and planning, and explore approaches that harness strengths such as energy/motivation, hyperfocus, peer support, and creativity. More broadly, larger-scale survey work with more representative samples of ED clinicians may be useful to better understand training needs, confidence levels, and service requirements in this area. Research should also assess training initiatives to improve ED clinicians’ confidence in recognising and working with ADHD.

## Conclusion

Clinician perspectives largely aligned with patient accounts, suggesting that ADHD-related disorganisation, reward and sensory seeking, and emotional volatility may underpin ED behaviours in populations with ADHD. However, quantitative evidence indicates that emotional dysregulation and reward processes may play a stronger mediating role than disorganisation, despite clinicians prioritising structure and planning in treatment. This may reflect how disorganisation disrupts engagement with therapy and can be misinterpreted as treatment resistance, while sensory seeking remains largely unexplored in quantitative research. Clinicians also described collaboratively adapting treatment by drawing on recognised ADHD strengths such as creativity, energy, and idea generation, with multidisciplinary input, particularly from occupational therapists and art therapists, seen as central. However, clinicians reported low confidence in recognising ADHD within ED services and uncertainty around medication decisions, highlighting the need for standardised screening and clearer guidance. Concerns about long ADHD service waiting times, limited consideration of EDs within ADHD assessments, and unclear prescribing responsibilities further emphasise the need for improved collaboration and knowledge exchange between ED and ADHD services.

## Supplementary Information

Below is the link to the electronic supplementary material.

Supplementary Material 1.

## Data Availability

The datasets generated during and analysed during the current study are available from the corresponding author for secondary analysis on request, as stipulated in participants’ consent forms.

## Abbreviations

ADHD: Attention-deficit/hyperactivity disorder
AN: Anorexia nervosa
ASRS: Adult ADHD Self-Report Scale
BED: Binge eating disorder
BN: Bulimia nervosa
ED: Eating disorder
EDNOS: Eating disorder not otherwise specified
