# Supplementary material for: Adapting eating disorder treatment for ADHD: clinicians’ perspectives on building structure, harnessing strengths, and negotiating boundaries
Source: J Eat Disord. 2026 Aug 19;14:268. doi: 10.1186/s40337-026-01740-5 (PMC13760909; doi:10.1186/s40337-026-01740-5)
Supplement: Supplementary file 1 [file 40337_2026_1740_MOESM1_ESM.docx]

**Adapting eating disorder treatment for ADHD: Clinicians’ perspectives on building structure, harnessing strengths, and negotiating boundaries**

***Supplementary Appendix 1. Interview guide for ADHD-related questions.***

1. Have you ever come across ADHD patients with EDs?
2. What do you notice about these patients?
3. What do you do if you think a patient has ADHD?
4. How do you judge the difference between ED symptoms and ADHD traits?
5. What adaptions do you make for ADHD patients?
6. Are there any particular challenges in working with ADHD patients with EDs?
7. Are there any advantages or strengths in working with ADHD patients with EDs?
8. What additional support would you find helpful in working with this group?
9. What do you think your service does well in working with ADHD patients with EDs? What could be improved?
10. Is there anything else you’d like to mention regarding ADHD?
